# A Case of Carcinoma Metastasis of Unknown Primary Mimicking Spondylodiscitis in an HIV+ Patient

**DOI:** 10.7759/cureus.67320

**Published:** 2024-08-20

**Authors:** Anıl Erol, Khassan Saidazimov, Mustafa Serdar Bölük, Taşkın Yurtseven, Hüseyin Biçeroğlu

**Affiliations:** 1 Department of Neurosurgery, Ege University Faculty of Medicine, Izmir, TUR

**Keywords:** spondylodiscitis, hiv-positive, unknown primary, cancer metastasis, carcinoma

## Abstract

In this case report, a case of carcinoma metastasis of unknown primary mimicking spondylodiscitis in a patient with acquired immunodeficiency syndrome (AIDS) is presented. A 50-year-old AIDS patient presented with a history of mechanical falls from his own level one month ago and leg weakness for the last three days. Spinal magnetic resonance imaging (MRI) revealed a compression fracture of the T4 vertebral body, spinal cord compression, and pathology compatible with spondylodiscitis. Posterior decompression and fusion were performed, and the patient benefited. The preoperative ASIA score was C, and the postoperative ASIA score was D. The sample taken from the lesion for pathology showed carcinoma metastasis. Tumor markers and whole-body computed tomography (CT) and MRI results did not support primary malignancy. Positron emission tomography was planned for further evaluation but could not be performed due to the poor general condition of the patient. During follow-up, the patient died of sepsis due to an intensive care unit infection. As new cases of carcinoma metastasis mimicking spondylodiscitis in AIDS patients are added to the literature, we will have more information about the diagnosis and treatment process.

## Introduction

Cancers of unknown primary origin (CUP) are tumors whose primary focus cannot be identified despite anamnesis, physical examination, various laboratory findings, and imaging methods [1]. CUP is seen between 3% and 5% worldwide and is the 6th to 8th most common cancer [2,3]. It is the 3rd to 4th most common cause of cancer death [3]. 

There are various hypotheses regarding etiology and pathogenesis. Simultaneous division of premalignant or malignantly transformed stem cells may produce daughter cells that do not proliferate locally but can metastasize [4]. Although there is no tumor development in the primary tissue, it may spread to another region [4]. The majority of CUP patients (80-85%) have a poor prognosis [2]. The mean age at diagnosis is 60 years, and the incidence is slightly higher in men [5]. In less than 20% of cancer patients with unknown primary sites, the primary site of cancer is detected before death. Autopsy studies have reported that 70% of cases remain undiagnosed [4].

There are studies suggesting the use of PET/CT in finding the primary focus. In a meta-analysis, the rate of 18F-FDG PET/CT to detect the primary tumor was calculated at 37% [6].

In this article, we report a case of thoracic metastasis of unknown primary origin in an HIV-positive patient who was diagnosed with carcinoma metastasis on pathology, while preoperative radiologic imaging suggested spondylodiscitis.

## Case presentation

**Figure 1:**
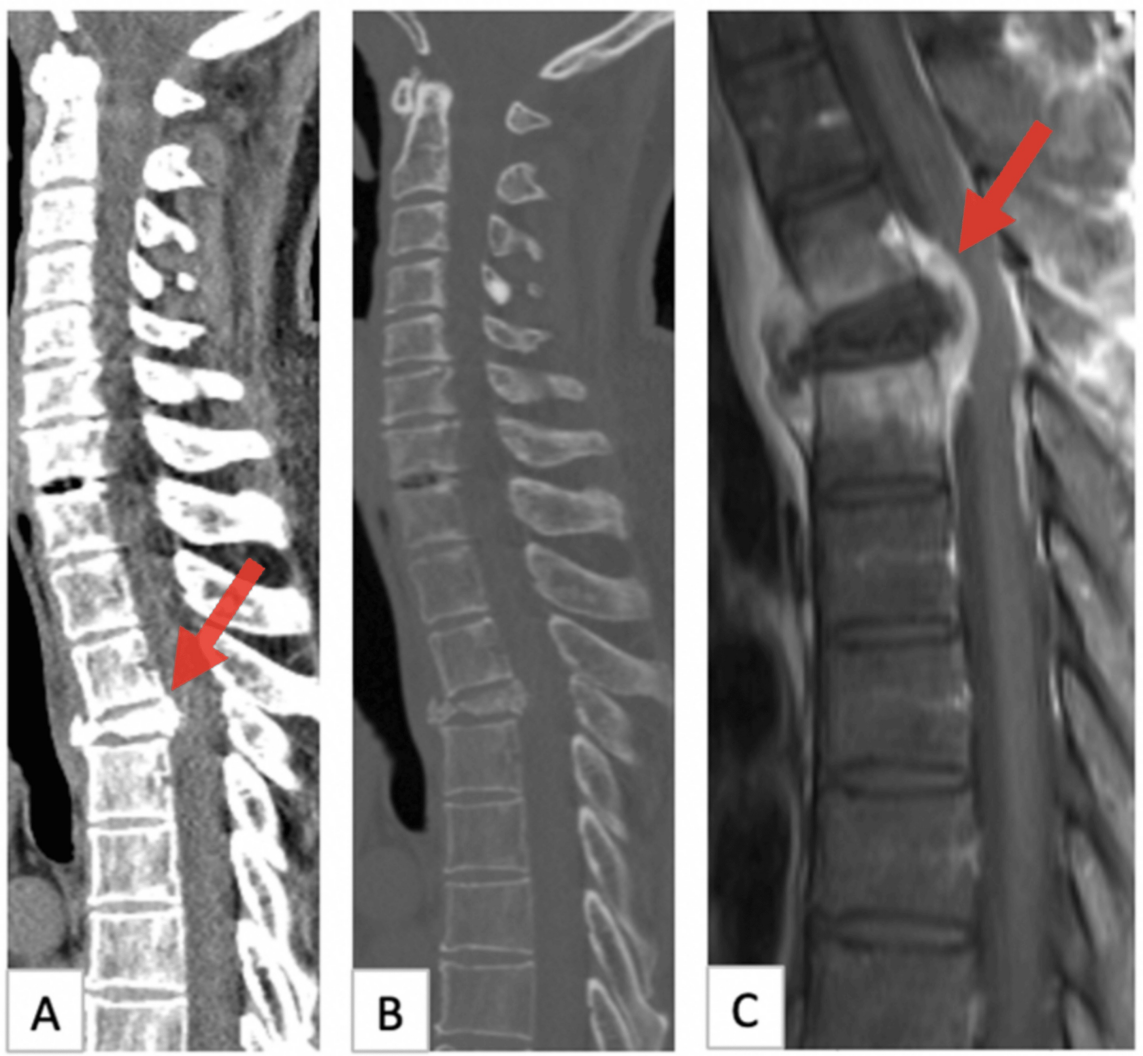
Preoperative images of the patient A, B: Sagittal CT showed compression fracture of four thoracic vertebrae and compression of the spinal canal, C: MR sequence after sagittal contrast showed contrast enhancement of the mass and compression of the spinal canal. T4 vertebral body compression fracture, spinal cord compression (arrow).

A 50-year-old AIDS patient presented with a history of mechanical falls from his own level one month ago and leg weakness for the last three days. Spinal magnetic resonance imaging (MRI) revealed a compression fracture of the T4 vertebral body, spinal cord compression, and pathology compatible with spondylodiscitis (Figure 1). 

**Figure 2:**
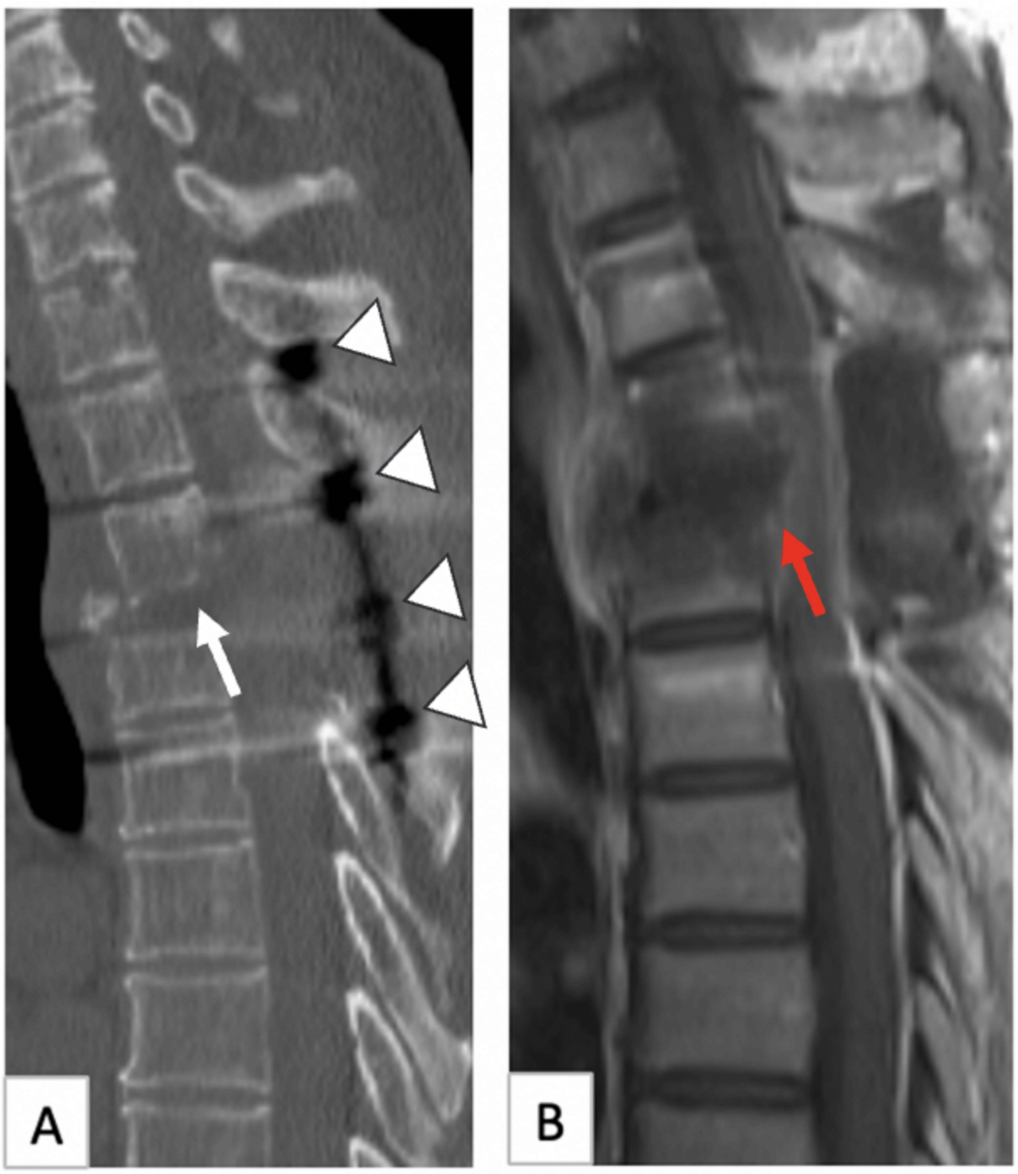
Postoperative images of the patient A: Sagittal CT shows that the fourth thoracic vertebra with compression fracture was removed by corpectomy (white arrow) and instrumented (arrowhead). B: Sagittal post-contrast MR sequence demonstrates reduction of edema and resection of the mass (red arrow).

Posterior decompression and fusion were performed, and the patient benefited (Figure 2). The preoperative ASIA score was C, and the postoperative ASIA score was D. 

The sample taken from the lesion for pathology showed carcinoma metastasis. Tumor markers and whole-body computed tomography (CT) and MRI results did not support primary malignancy. Positron emission tomography was planned for further evaluation but could not be performed due to the poor general condition of the patient.

**Figure 3:**
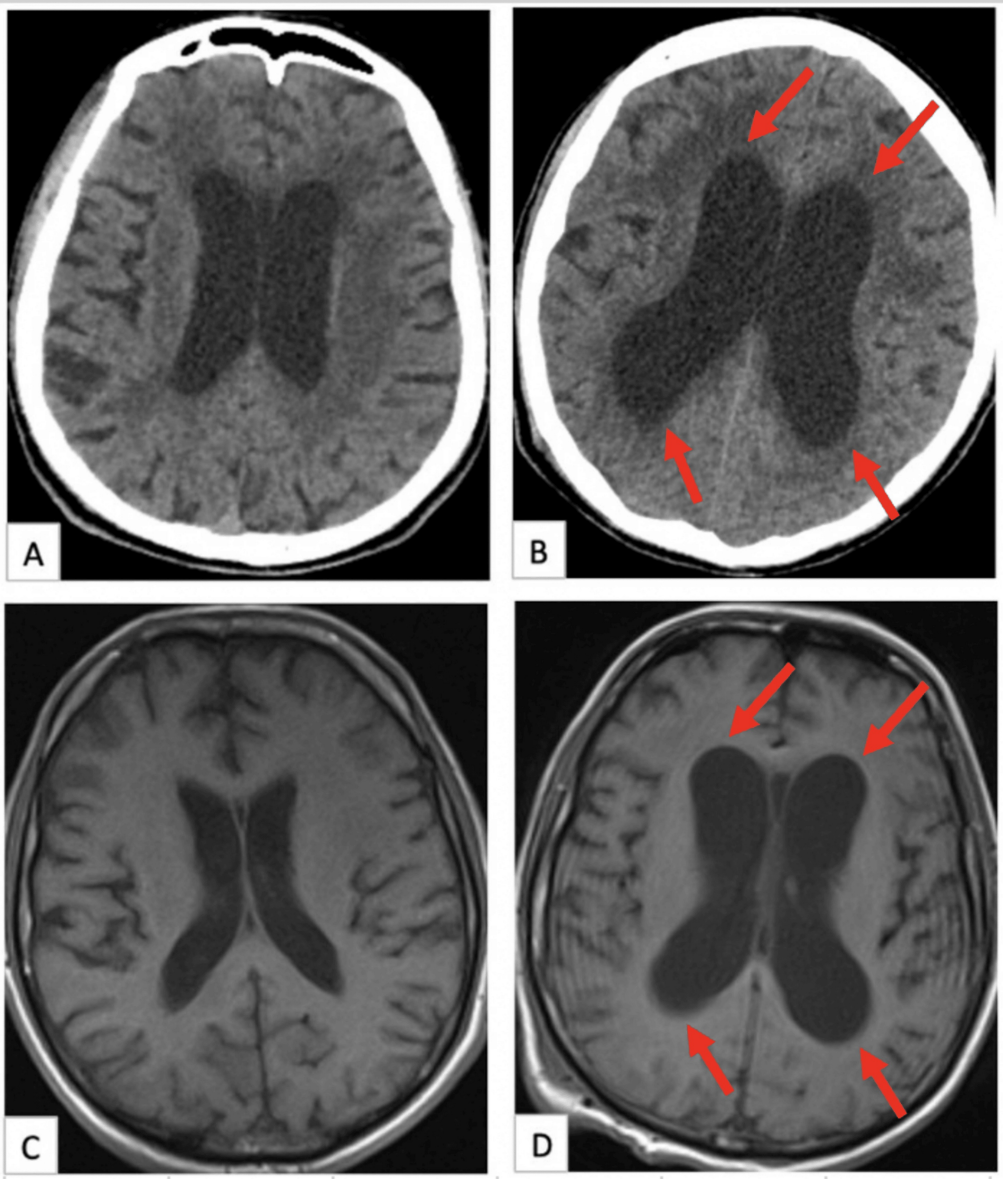
Cranial images of the patient A: Cranial CT before surgery, B: Cranial CT showing the development of hydrocephalus during follow-up, C: Cranial MRI before surgery, and D: Cranial MRI showing the development of hydrocephalus during follow-up. Red arrows show that ventricle sizes are more dilated.

The patient developed hydrocephalus while being monitored in the intensive care unit (Figure 3). A cranial MRI scan showed findings suggestive of ventriculitis, prompting the initiation of external ventricular drainage and appropriate antibiotic therapy. *Acinetobacter baumannii* was isolated in the cerebrospinal fluid culture along with the detection of Cytomegalovirus (CMV) positivity. As subsequent cerebrospinal fluid cultures did not show any growth, a ventriculoperitoneal shunt was placed in the patient. During follow-up, the patient died of sepsis due to an intensive care unit infection.

## Discussion

The general approach in patients with fractures of the thoracic vertebrae due to metastatic mass and spinal cord compression is to relieve and stabilize the cord compression to alleviate symptoms and relieve pain. In our case, we performed mass excision and laminectomy to relieve spinal cord compression and then stabilized it using instrumentation.

The pathology report of our case was concluded to be carcinoma metastasis, but the primary site of carcinoma was not clear from the pathology data. It was learned from the pathology data that the primary carcinoma metastasis might have originated from the urogenital system. However, further investigations, including tumor marker tests and imaging studies, did not provide any evidence supporting urogenital carcinoma.

Furthermore, a whole-body CT scan revealed only millimetric lesions in the lungs that could indicate a mass or infection. A PET-CT scan was planned for further evaluation but unfortunately could not be performed due to the patient's poor general health.

In our literature search focusing on carcinoma metastases mimicking spondylodiscitis using the PubMed database, we found two related cases [7,8]. In these cases, the initial diagnosis was spondylodiscitis, but pathology revealed carcinoma metastases. In contrast, in another reported case [9], carcinoma metastasis was initially suspected, but pathology findings showed spondylodiscitis. 

A review of the available literature on metastases of unknown primary origin in HIV-positive patients revealed very limited data. In an article in which four HIV-positive patients with lung adenocarcinoma metastasis of unknown primary were presented, it was stated that there were no previously published data on carcinomas of unknown primary in HIV+ patients [10]. This situation underlines the need for further studies in this field. 

## Conclusions

Carcinoma metastasis mimicking spondylodiscitis in AIDS patients is a rare condition, and patients diagnosed with carcinoma metastasis should be evaluated with advanced investigations such as whole-body CT, MRI, and PET, and the primary focus should be investigated. Due to the poor general condition of our patient, we could not perform advanced examinations, but as new cases are added to the literature, we will have more information about the diagnosis and treatment process.

In conclusion, this case shows that carcinoma metastasis should be kept in mind in the differential diagnosis of spondylodiscitis in AIDS patients.
